# Autistic Traits Among Adolescents and Young Adults Under Assessment for Psychiatric Conditions: An Experimental Analysis of Prevalence – CORRIGENDUM

**DOI:** 10.1192/bjo.2024.40

**Published:** 2024-04-03

**Authors:** Sanem İnci, Veronica Nisticò, Irene Folatti, Giulia Santangelo, Claudio Sanguineti, Raffaella Faggioli, Angelo Bertani, Orsola Gambini, Benedetta Demartini

This abstract was inadvertently published without the inclusion of Veronica Nisticò, Irene Folatti, Giulia Santangelo, Claudio Sanguineti, Raffaella Faggioli, Angelo Bertani, Orsola Gambini, Benedetta Demartini as authors. This has now been updated and this corrigendum published.
